# Comparing Sensitivity of Ultrasonography and Plain Chest Radiography in Detection of Pneumonia; a Diagnostic Value Study

**Published:** 2019-01-22

**Authors:** Ebrahim Karimi

**Affiliations:** 1. Emergency Department, Be’sat Hospital, AJA University of Medical Sciences, Tehran, Iran.

**Keywords:** Diagnostic imaging, ultrasonography, sensitivity and speciﬁcity, radiography, thoracic, tomography, x-ray computed

## Abstract

**Introduction:**

Rapid detection of pneumonia and early initiation of antibiotic therapy are associated with better prognosis in patients. The present study was designed aiming to evaluate the sensitivity of chest ultrasonography performed by emergency medicine specialists in detection of pneumonia and comparing it with plain radiography.

**Methods:**

In the present diagnostic accuracy study, patients presenting to the emergency department with clinical symptoms of lung infection underwent plain radiography, ultrasonography, and computed tomography (CT) scan of chest and the screening performance characteristics of plain radiography and ultrasonography were compared considering CT scan findings as the gold standard.

**Results:**

280 patients with the mean age of 56.47 ± 19.79 (10 – 92) years were studied (57.1% male). The results of chest CT scan were indicative of infection symptoms being present and confirmed pneumonia diagnosis for all the patients. Out of the 280 cases of pneumonia confirmed via chest CT scan, 17 (6.1%) cases were not detected via ultrasonography and 48 (17.1%) cases were missed by chest radiography (false negative cases). No false positive case was reported by ultrasonography or chest x-ray. Since all of the CT scans were positive, no comment can be made regarding the specificity of the evaluated tests, but sensitivity of ultrasonography and plain radiography were 93.92 (90.28 – 96.31) and 82.85 (77.81 – 86.97), respectively (p = 0.583).

**Conclusion:**

Based on the findings of the present study, although the sensitivity of ultrasonography in detection of pneumonia was significantly higher than chest x-ray, overall the screening performance characteristics of the 2 tests were not significantly different. Therefore, considering characteristics such as safety, low cost, being portable, and being available, ultrasonography seems to be a reasonable tool for screening and diagnosis of patients with pneumonia.

## Introduction:

Pneumonia is a common cause of emergency department (ED) visits and has been reported to be responsible for 1 million hospitalizations annually (1). Patients usually present with cough, tiredness, fever, shortness of breath, and pleuritic chest pain in typical cases.

Diagnosis of pneumonia is usually done in ED and if it is not detected it will be associated with a high risk of complications and mortality. Rapid diagnosis of pneumonia and early treatment with antibiotic are factors that have been associated with better prognosis and outcome (2, 3).

History taking and clinical examination are the basis of diagnosing this disease and there are positive findings in history and clinical examination of these patients, including rales, rhonchi, wheezing, bronchial respiratory sounds, reduction of respiratory sounds, and dullness to percussion in chest (4). Confirmation of diagnosis for this disease depends on imaging. Sensitivity of plain chest radiography in detection of pneumonia has been reported as 38% to 76% in studies (5-8). Although chest computed tomography (CT) scan is considered the standard in detection of pneumonia, it is associated with disadvantages such as unavailability and high exposure to radiation (9, 10). Currently, chest ultrasonography is very popular for detection of pulmonary diseases such as pneumothorax, pulmonary edema, pleural effusion and pneumonia as a cheap, available, safe, and portable tool (11). Sensitivity of ultrasonography compared to radiography has been reported to be between 80% and 90% in detection of pneumonia (8, 12, 13). With the introduction of emergency medicine discipline in Iran, all the teaching wards have been provided with ultrasound devices and emergency medicine specialists have been trained for using this tool. Therefore, the present study was designed aiming to evaluate the sensitivity of chest ultrasonography performed by emergency medicine specialists in detection of pneumonia and comparing it with plain radiography.

## Methods:

***Study design and setting***

The present diagnostic accuracy study has been performed on patients presenting to the emergency department of Be’sat Hospital, Tehran, Iran, with symptoms of pulmonary infection in the time interval between October 2015 and March 2017. Methodology of the present study was approved by the ethics committee of AJA University of Medical Sciences and the researchers adhered to the ethical principles presented in Helsinki declaration and keeping patient data confidential. All the probable costs inflicted on the patients were provided by the research team.

***Participants***

Adult patients (over 18 years of age) presenting to the mentioned emergency department with clinical symptoms of pneumonia such as cough, phlegm, shortness of breath, hemoptysis, and temperature higher than 38°C were studied using non-probability convenience sampling. Pregnant patients, those with immunodeficiency, those consuming corticosteroids or any other immunosuppressant, those affected with chronic kidney or liver failure, and hemodynamically unstable patients were excluded from the study. No sex limitation was applied in the present study.

***Imaging***

After history taking, accurate clinical examination, evaluation of vital signs, dismissing cardiac causes for shortness of breath including heart attack, and providing primary healthcare, explanations regarding the aims of the study were given to the patients or their relatives. Then chest ultrasonography was performed on participants by trained emergency medicine residents under supervision of the in charge attending emergency medicine specialist.

All ultrasounds were performed with Samsung HM70A device using a curved probe with 3.5 – 5 MHz frequency for observing the pulmonary and pleural parenchyma and 7.5 – 10 MHz frequency for observing more superficial lesions of the chest. For ultrasonographic examination of the lungs, each half of the chest was divided into anterior (from the parasternal line to the anterior auxiliary line), lateral (between the posterior and middle auxiliary lines), and posterior (from the posterior auxiliary line to the paravertebral line) sections and evaluated separately. The depth of ultrasound field in this study was adjusted between 16 and 18 centimeters. Ultrasonographic symptoms evaluated in this study included air bronchogram, fluid bronchogram, pleural effusion, b lines (comet tail sign), and subpleural consolidation.

After the performance of ultrasonography, patients were sent to the imaging unit to undergo plain radiography and chest CT scan. Chest radiographies were performed in standing position from both anterior-posterior and lateral views for all the patients. Helical CT scan was performed on patients without injection of a contrast agent.

Interpretation of the chest radiographies and CT scans was done by an emergency medicine specialist and a radiologist, separately, both of whom were blind to the clinical and ultrasonography findings of the patients.

***Data gathering***

An emergency medicine specialist was responsible for recording patients’ data. For this purpose, a checklist was filled out for each patient, which consisted of age, sex, chest ultrasound findings, plain chest radiography findings, and chest CT scan findings.

***Statistical analysis***

To analyze data, SPSS 20 statistical software was used. Findings were reported using mean and standard deviation or frequency and percentage indices summarized in tables. To calculate screening performance characteristics of ultrasonography and plain chest x-ray, a medical calculator was used and sensitivity, specificity, positive and negative predictive values (PPV and NPV), and positive and negative likelihood ratios (PLR and NLR) were reported with 95% confidence interval (CI). P values less than 0.05 were considered statistically significant.

## Results:

280 patients with the mean age of 56.47 ± 19.79 (10 – 92) years were studied (57.1% male). Table 1 shows the chest ultrasonography findings of these patients. In 118 (42.1%) cases, positive ultrasonography findings were on the right, 96 (34.3%) were on the left, and 48 (17.1%) were bilateral. The results of chest CT scan were indicative of infection symptoms being present and pneumonia diagnosis was confirmed for all the patients. Out of the 280 cases of pneumonia confirmed via chest CT scan, 17 (6.1%) cases were not detected via ultrasonography and 48 (17.1%) cases were missed by chest radiography (false negative cases). No false positive case was reported by ultrasonography or chest x-ray. Table 2 depicts the screening performance characteristics of ultrasonography and plain chest radiography in detection of pneumonia considering chest CT scan as the gold standard. Since all of the CT scans were positive, no comment can be made regarding the specificity of the evaluated tests, but as shown in table 2, sensitivity of ultrasonography in detection of pneumonia was higher than plain radiography, yet this difference was not clinically significant (p = 0.583).

## Discussion

Based on the findings of the present study, although the sensitivity of ultrasonography in detection of pneumonia was significantly higher than chest x-ray, overall the screening performance characteristics of the 2 tests were not significantly different. Therefore, considering characteristics such as safety, low cost, being portable, and being available, ultrasonography seems to be a reasonable tool for screening and diagnosis of patients with pneumonia. 

**Table 1:** Chest ultrasonography findings of studied patients

**Signs**	**Frequency (%)**
Air bronchogram	160(57.1)
Fluid bronchogram	118 (42.1)
Pleural effusion	143 (51.1)
B lines (comet tail sign)	126 (45.0)
Subpleural consolidation	62 (22.1)

**Table 2:** Screening performance characteristics of chest ultrasonography and radiography in diagnosis of pneumonia

**Characteristics**	**Ultrasonography**	**Radiography**
**Sensitivity**	93.92 (90.28-96.31)	82.85 (77.81-86.97)
**Specificity**	NaN	NaN
**PPV**	100.00 (98.20- 100.00)	100.00 (97.96- 100.00)
**NPV**	0.00 (0.00-22.92)	0.00 (0.00-20.30)
**PLR**	Infinite	Infinite
**NLR**	Infinite	Infinite

NaN: the calculation cannot be performed because the values entered include one or more instances of zero.

PPV: positive predictive value; NPV: negative predictive value; PLR: positive likelihood ratio; NLR: negative likelihood ratio

Using lung ultrasonography in evaluation of patients is becoming more popular in emergency department (14). Although old methods are still used for diagnosis, lung ultrasonography can reduce the diagnostic errors of plain radiography as a helping tool.

In addition, ultrasound is a useful device for follow-up of patients with pneumonia. Decrease in air broncogram and the volume of pleural effusion compared to the primary ultrasounds can be considered signs of improvement in the patient (15). 

In children, due to the high risk of cancer in the early years of life in case of being exposed to radiation, ultrasonography can be a better method than CT scan and plain chest radiography for detection of pneumonia. In children, due to the small body size and small pulmonary mass, echo penetration is higher and a higher volume of the lung can be seen (15).

By studying 144 adults, Bourcier et al. reported 95% sensitivity for ultrasonography in detection of pneumonia and introduced it as the first line of diagnosis for these patients (5).

In 2012, by studying 362 patients with suspected pneumonia acquired from the society, Reissig et al. reported 93% sensitivity for detection of pneumonia and expressed that about 8% of the lesions related with pneumonia are not detectable via ultrasonography and negative results in ultrasonography are not enough for ruling out pneumonia (15).

In a brief report, Taghizadieh et al. reported the high accuracy of ultrasonography compared to plain chest radiography in detection of pneumonia (7).

Of course, ultrasonography is dependent on the individual performing it and emergency physicians need to be familiar with the ultrasonographic appearance of other differential diagnoses that can lead to consolidation such as lymphoma and bronchoalveolar carcinoma (16, 17).

Overall, like many other diagnostic methods, if ultrasonography is performed for a suitable person, in proper conditions and by a skillful person, it can play an important role in screening and detection of pneumonia, which lead to rapid initiation of treatment, improving the outcome of patients with pneumonia as a result. Therefore, including ultrasonography training in detection of pneumonia in emergency resident’s course syllabus seems important more than ever.

Limitations

The present study was done on patients with suspected pneumonia in one center and this limits the generalization of its findings. On the other hand, the dependence of ultrasonography on the individual performing it should be considered in interpretation and generalization of the data.

## Conclusion:

Based on the findings of the present study, although the sensitivity of ultrasonography in detection of pneumonia was significantly higher than chest x-ray, overall the screening performance characteristics of the 2 tests were not significantly different. Therefore, considering characteristics such as safety, low cost, being portable, and being available, ultrasonography seems to be a reasonable tool for screening and diagnosis of patients with pneumonia. 
